# A Novel Hydrophilic Colloidal Polysaccharide from *Rosa roxburghii* Tratt: Structural Characterization, Rheological Behavior and Immunomodulatory Activity

**DOI:** 10.3390/foods15101641

**Published:** 2026-05-08

**Authors:** Chenxi Cui, Miao Wang, Qiuli Zhang, Xinzhu Zhang, Qi Zhu, Liuya Wang, Tengda Li, Zhenyuan Zhu

**Affiliations:** 1 State Key Laboratory of Food Nutrition and Safety, Tianjin University of Science and Technology, Tianjin 300457, China; 18506469377@163.com (C.C.); 13821701321@163.com (M.W.);; 2 Key Laboratory of Food Nutrition and Safety, Ministry of Education, Tianjin University of Science and Technology, Tianjin 300457, China; 3 College of Food Science and Engineering, Tianjin University of Science and Technology, Tianjin 300457, China

**Keywords:** *Rosa roxburghii*, acidic polysaccharide, viscosity, immunoactivity, structural characterization

## Abstract

*Rosa roxburghii* Tratt is recognized as an edible and medicinal plant valued for its nutritional and medicinal properties. Polysaccharides are among its key bioactive constituents. A homogeneous polysaccharide, designated RTW-1, was extracted and purified from the fruit of *Rosa roxburghii* Tratt. Its molecular mass was determined by HPLC to be 2.16 × 10^3^ kDa. Monosaccharide composition and methylation analysis showed that RTW-1 is mainly composed of glucose, arabinose, and galacturonic acid in a molar ratio of 1.00:0.48:0.74. The uronic acid content was measured as 55.21%, and the degree of esterification was 58.30%. The glycosidic linkages identified included (→2,3,4)-Man*p*-(1→, →4)-Ara*p*-(1→, →4)-GalA*p*-(1→, T-Rha*p*, →4)-Glc*p*-(1→, and (→2,3,4)-Xyl*p*-(1→). Shear-thinning behavior was revealed by rheological analysis. At 30 mg/mL, the thixotropic loop area reached 143.8 Pa/s, which was 20 times higher than that at 12 mg/mL. In RAW264.7 macrophages, cell proliferation was promoted by RTW-1. At 80 μg/mL, phagocytic activity was increased by 88%, and NO production was enhanced by 3.1-fold. Concentration-dependent upregulation of TNF-α, IL-6, IL-1β, and IL-10 mRNA expression was observed by qRT-PCR, with maximum increases of 3.2-, 4.1-, 2.8-, and 2.5-fold, respectively. In conclusion, RTW-1 possesses favorable gel-forming and immunostimulatory properties and shows potential for promoting intestinal immune activity, suggesting its promise as a functional food ingredient.

## 1. Introduction

Cancer (malignant tumors) is a common disease worldwide, and its incidence continues to rise, posing a serious threat to human health [1]. Chemotherapy remains one of the primary treatments for cancer because it can suppress tumor growth and metastasis. However, as a systemic therapy, it damages normal cells and causes toxic side effects, including immunosuppression, gastrointestinal reactions, leukopenia, and bone marrow suppression [2]. Cyclophosphamide (Cy), a widely used chemotherapeutic agent, shows efficacy in treating malignancies but also leads to significant side effects such as immunosuppression, intestinal mucosal damage, reproductive toxicity, and hepatotoxicity [3]. High-dose Cy impairs the gastrointestinal mucosa, increasing the risk of immunodeficiency and secondary infections. Consequently, to reduce Cy’s side effects and improve treatment outcomes, efforts are underway to develop dietary immunomodulators that support intestinal mucosal immunity. Recent studies have further demonstrated that natural polysaccharides can protect against chemotherapy-induced intestinal mucositis; for example, *Hypsizygus marmoreus* polysaccharides (HMPs) were shown to mitigate cisplatin-induced gastrointestinal injury and immune dysfunction while promoting gut microbiota balance [4].

The immune system is a crucial defense mechanism that protects the body against pathogens and maintains immune homeostasis. The gut, as the largest digestive and immune organ, features a defensive barrier formed by tightly connected intestinal epithelial cells (IECs) to prevent microbial invasion [5]. This barrier hosts various immune cells, such as T cells, B cells, innate lymphoid cells (ILCs), and cells of the mononuclear phagocyte system. Growing evidence indicates that natural polysaccharides can enhance immunity and strengthen intestinal barrier function by modulating the gut microbiota [6]. Studies show that polysaccharides from *Bacillus subtilis*, *Lycium*, and Polygonatum can modulate gut microbiota and enhance intestinal immunity. B. subtilis polysaccharides promote specific bacteria while suppressing others to regulate colonic mucosal immunity [7]. *Lycium* polysaccharides increase the abundance of beneficial bacterial families associated with immune function [8]. *Polygonatum* polysaccharides help protect the intestinal barrier and alleviate colonic damage by regulating microbial diversity [9]. Notably, Chinese yam polysaccharide (CYP) was recently reported to enhance jejunal structural development and mucosal immunity via activation of the MAPK ERK1/2–Nrf2 signaling pathway [10].

Viscosity is a key rheological indicator of polysaccharide solutions, closely related to molecular weight, esterification degree, and branching degree. Pectin is a multifunctional polysaccharide whose structural characteristics, including the degree of esterification (DE), molecular weight (MW), and branching pattern, directly influence its gelling, emulsifying, and biological activities [11,12]. At acidic pH levels, such as those found in the stomach and small intestine, high-methoxyl pectin (HMP) can more effectively retain fat and bile salts, thereby contributing to improved cholesterol regulation [13]. In vitro dynamic digestion simulations indicate that pectin resists enzymatic degradation, facilitating prolonged retention in the gut until it reaches the colon for microbial fermentation [14]. This “viscosity–gut interaction–immunomodulation” relationship suggests that high-viscosity polysaccharides may exert superior effects in the intestine due to their rheological properties, providing critical evidence for the screening and application of functional polysaccharides in the future.

*Rosa roxburghii* Tratt, the fruit of a deciduous shrub in the *Rosaceae* family, has garnered significant attention for its sweet, sour, and astringent flavor profile, earning it the title “King of Vitamin C” among fruits. In China, it is widely utilized as both a food and medicinal resource, representing a nutrient-dense fruit with combined health-promoting and therapeutic properties [15]. Previously regarded as an “anti-cancer treasure fruit,” long-term intake of *Rosa roxburghii* concentrated juice by individuals who smoke or consume alcohol may help reduce cancer risk by suppressing the expression of tumor biomarkers such as 8-hydroxy-2′-deoxyguanosine and carcinoembryonic antigen (CEA) [16]. Between 2025 and 2026, significant progress has been made in research on polysaccharides from *Rosa roxburghii* Tratt. In terms of structural characterization, Wan et al. isolated cell wall polysaccharides from the fruit using a sequential extraction strategy. They identified two pectin fractions, designated WSP and CSP [17]. Both fractions are characterized by a high degree of methylesterification and are rich in homogalacturonan (HG) domains. Jiao et al. systematically compared four extraction methods for *R. roxburghii* polysaccharides: acid extraction, enzyme-assisted extraction, ultrasound-assisted extraction, and ultrasound-assisted enzyme extraction [18]. They examined the effects of these methods on rheological behavior. The polysaccharides extracted by acid and ultrasound-assisted methods exhibited pseudoplastic fluid behavior. Chen et al. demonstrated that RTFPs significantly enhanced the phagocytic capacity of RAW264.7 macrophages (by 12.61–76.63%) and stimulated NO and TNF-α secretion [19]. These effects were observed within a concentration range of 100–400 μg/mL. The authors attributed the bioactivity to the high uronic acid content [20]. However, further research is still needed to elucidate the mechanisms through which Rosa roxburghii exerts its functional effects. A deeper understanding of its action pathways is crucial for enhancing its added value and maximizing its potential efficacy. This remains an urgent issue to be addressed by both researchers and the industry at present [21]. In this study, we isolated a homogeneous acidic polysaccharide (RTW-1) from *R. roxburghii*. This polysaccharide has an exceptionally high molecular weight. It is significantly higher than any previously reported components. RTW-1 also has a distinct glycosidic linkage pattern, including mannose- and xylose-rich linkages that have not been documented before. In addition, it shows characteristic shear-thinning rheological behavior. We also evaluated its concentration-dependent immunomodulatory activity in RAW264.7 macrophages. Therefore, this work provides a new polysaccharide candidate with distinctive structural and functional features for potential use in functional foods.

## 2. Materials and Methods

### 2.1. Experimental Materials

*Rosa roxburghii* Tratt (Chinese rosehip) was sourced from Qixingguan District, Bijie, Guizhou Province, China. DEAE-52 and Sephadex G-150 were provided by Beijing Ruida Huihui Technology Co., Ltd. (Beijing, China). Standard monosaccharides, including D-galactose, D-glucose, D-mannose, D-xylose, L-arabinose, L-rhamnose, D-glucuronic acid, and D-galacturonic acid, were supplied by Sigma-Aldrich Corporation, St. Louis, MO, USA. T-series dextrans (T10, T40, T70, T110, T1000, T2000), Dulbecco’s Modified Eagle Medium (DMEM), CCK-8, acid phosphatase, lysozyme, superoxide dismutase, lipopolysaccharide, Escherichia coli serotype O55:B5, and NO assay kits were purchased from SolarBio Co., (Beijing, China). The Omega RNA extraction kit was purchased from SolarBio Co.

### 2.2. Isolation and Extraction of RTW

Optimal extraction conditions for crude *Rosa roxburghii* Tratt polysaccharides were determined through single-factor and response surface experiments. The extracted polysaccharides were precipitated with 80% ethanol, allowed to stand overnight and then concentrated by rotary evaporation. The resulting polysaccharide solution was mixed with Sevag reagent (n-butanol:chloroform = 1:4) at a 4:1 ratio. The mixture was vigorously shaken to denature and remove proteins. Centrifugation (4000 r/min, 15 min) was performed to separate and remove the protein layer, and the polysaccharide solution was retained. This solution underwent 100 kDa flow dialysis, and the collected dialysate was freeze-dried to obtain crude *Rosa roxburghii* Tratt polysaccharide RTW. To further purify RTW, DEAE-52 cellulose (Beijing Ruida Huihui Technology Co., Ltd. Beijing, China) and Sephadex G-150 column (Beijing Ruida Huihui Technology Co., Ltd. Beijing, China) chromatography were used in sequence. First, 50 mg of RTW was dissolved in 1 mL of distilled water and loaded onto a DEAE-52 cellulose ion-exchange column. Stepwise elution was carried out with 0, 0.1, 0.2, and 0.3 mol/L NaCl at a flow rate of 1 mL/min. Fractions were collected every 6 min, with 20 fractions per gradient, to separate polysaccharides according to their surface charge. Sugar content in the collected fractions was measured using the phenol–sulfuric acid method. The fraction showing the highest absorbance was then analyzed by high-performance liquid chromatography (HPLC) to confirm its homogeneity. This homogeneous fraction was further purified on a Sephadex G-150 gel filtration column. The purified polysaccharide fraction from *Rosa roxburghii* Tratt was designated RTW-1 and used for subsequent experiments.

### 2.3. Study on the Physicochemical Properties of RTW-1

#### 2.3.1. Determination of Polysaccharide Content

A 0.1 mg/mL solution of RTW-1 was prepared. Determine the sugar content of RTW-1 using the phenol–sulfuric acid method and plot a standard curve [22]. Calculate the sugar content in the sample based on the total sugar standard curve.

#### 2.3.2. UV Full-Wavelength Scan of RTW-1

A 1 mL volume of RTW-1 solution at a concentration of 1 mg/mL was prepared. Detect using a UV–visible spectrophotometer over the range of 190–400 nm [23].

#### 2.3.3. Polysaccharide Molecular Weight Determination

Weigh 1 mg each of the dextran series standards (T10, T40, T70, T110, T1000, and T2000) (SolarBio Co., Beijing, China) and the RTW-1 polysaccharide sample. Dissolve them completely in deionized water to prepare 1 mg/mL solutions. Determine the retention times of the standards with different molecular weights and the RTW-1 sample. Using the retention times and the calibration curve constructed with the molecular weights of the various dextran standards, substitute the retention time of RTW-1 into the calibration curve to calculate its molecular weight.

#### 2.3.4. Determination of Uronic Acid Content

The uronic acid content of RTW-1 polysaccharide from *Rosa roxburghii* Tratt was determined using the m-hydroxydiphenyl method [24]. A 0.1 mg/mL solution of RTW-1 was prepared, and the results were substituted into the standard curve equation to calculate the uronic acid content.

#### 2.3.5. Determination of Esterification Degree

According to the method described by Yu et al. [25], 200 mg of RTW-1 was accurately weighed into a conical flask. The sample was dissolved in 20 mL of ultrapure water, and 5 drops of phenolphthalein indicator were added. The solution was titrated with 0.1 mol/L NaOH standard solution until a stable pale red color persisted for 30 s without fading. Record the volume of base consumed (V_1_). Add 10 mL of the same concentration NaOH standard solution, shake at 40 °C for 4 h, then neutralize with 10 mL of 0.1 mol/L HCl solution. After thorough mixing, add phenolphthalein indicator (5 drops) again and continue titrating with the standard NaOH solution to the same endpoint. Record the volume V_2_. Perform three parallel replicates per group. Substitute the results into the calculation formula as follows:

DE = V_2_/(V_1_ + V_2_);

DE: Degree of esterification;

V_1_: Volume of NaOH consumed in the first titration;

V_2_: Volume of NaOH consumed in the second titration.

### 2.4. Structural Analysis of RTW-1

#### 2.4.1. Fourier Transform Infrared Spectroscopy (FT-IR) Analysis

The potassium bromide (KBr) pellet method was employed: 1 mg of RTW-1 was mixed with 150 mg of pure KBr powder, ground, and then pressed into a 1 mm thick transparent test pellet using a pellet press at 10 MPa. The sample was placed in the infrared spectrometer’s sample chamber. A cumulative scan of 32 passes over the 4000–500 cm^−1^ range yielded the characteristic infrared absorption spectrum of RTW-1.

#### 2.4.2. Congo Red Assay

Two milliliters of a 0.5 mg/mL RTW-1 solution are prepared and thoroughly mixed with 2 mL of a 50 μmol/L Congo Red solution. Add varying volumes of 1 mol/L NaOH solution to achieve final NaOH concentrations of 0, 0.05, 0.1, 0.15, 0.2, 0.25, 0.3, 0.35, and 0.40 mol/L in the reaction system. After 10 min of reaction, perform a full wavelength scan from 400 to 600 nm, determine the maximum wavelength, and record the results.

#### 2.4.3. Monosaccharide Composition Analysis

##### Polysaccharide Sample Degradation

Following the method described by Zhu et al. [26], 15 mg of RTW-1 was hydrolyzed with 2 mL of trifluoroacetic acid (TFA) at 110 °C for 2 h. The hydrolysate was dried under a flow of N2, after which 1 to 2 mL of methanol was added. The washing and drying steps were repeated five times.

##### Determination of Monosaccharide Composition in Acid Polysaccharides

Standard samples (L-Rha, D-Xyl, D-Glc, D-Gal, D-Man, L-Ara, D-GlcA, and D-GalA) (Sigma-Aldrich Corporation, St. Louis, MO, USA) and the degraded polysaccharide sample RTW-1 were weighed and placed into stoppered test tubes. Add 100 μL of 0.5 mol/L Na_2_CO_3_ solution and react at 30 °C for 30 min. Add 20 mg NaBH_4_ (react for 1.5 h), then titrate with 25% acetic acid until no bubbles are produced. Elution was performed using a cation exchange column. The eluate was rotary evaporated to dryness, then reacted with pyridine and n-propylamine at 55 °C for 30 min. After drying under N_2_, 2 mL each of pyridine and acetic anhydride were added, thoroughly vortexed, and reacted overnight. After drying, dissolve in chloroform, quench with water, and collect the chloroform layer for GC-MS analysis.

#### 2.4.4. Methylation Analysis

##### Uronic Acid Reduction

Following the uronic acid reduction method described by Hao et al. [27], dissolve 20 mg of RTW-1 in 5 mL of ddH_2_O. Add 15 mg of the reaction catalyst, carbodiimide, and mix with 0.1 mol/L HCl solution to maintain the system pH at 4.75. Under magnetic stirring, adjust the pH to 7.0 using 5 mL of 0.1 mol/L NaBH_4_ solution. Subsequently, add 2 mol/L HCl to stabilize the pH at 4.0. After standing at room temperature, transfer the solution to a 3500 Da dialysis membrane for continuous dialysis and freeze-dry for later use.

##### Methylation Reaction

Dissolve the reduced product in 2 mL of anhydrous DMSO (dried using a 3A molecular sieve). Under a nitrogen atmosphere, add 25 mg of NaH powder. React in the dark at 18 °C with ultrasonic agitation for 30 min. Then add 2 mL of methyl iodide and react in the dark at 18 °C with ultrasonic agitation for 2 h. Nitrogen flushing and ultrasonication were repeated several times. Take a small sample for infrared spectroscopy analysis to determine whether the methylation reaction is complete. After methylation was complete, the reaction mixture was extracted with dichloromethane and water, and then dried under nitrogen [28].

##### Degradation and Reduction

Take fully methylated RTW-1 samples, add 2.0 mL trifluoroacetic acid (2 mol/L), hydrolyze at 110 °C for 3 h, cool, dry under nitrogen, rinse repeatedly with methanol solution, then dry again. Resuspend the product in ultrapure water, add 25.0 mg NaBH_4_, and shake overnight for reduction. Adjust the pH to 5.0 with 0.1 mol/L acetic acid solution, dry under nitrogen, add 2 mL methanol and one drop of acetic acid, and repeat the drying process five times to ensure complete removal of residual NaBH_4_ (omit acetic acid in the final step).

##### Derivatization Preparation

Add 2 mL of acetic anhydride to the obtained sample. Place it in a 100 °C oil bath for the acetylation reaction for 1 h. Allow it to cool to room temperature and then dry under a stream of nitrogen. Repeat methanol washing and drying three times. Extract, then perform GC-MS analysis.

#### 2.4.5. Scanning Electron Microscopy Analysis

Take an appropriate amount of RTW-1, adhere it to the silicon wafer, remove excess sample with a dust blower, gold-coat the sample, and observe.

### 2.5. Steady-State Rheological Testing

The experimental methodology was optimized based on studies by Lin et al. [29] and Zhao et al. [30]. All rheological experiments were conducted on a dynamic rheometer (HAAKEMARS 60, Thermo Fisher Scientific, Braunschweig, Germany). Rotor C35 1°/Ti-02160374 was used with a gap maintained at 0.053 mm.

The steady-state flow behavior of RTW-1 solutions at various concentrations was measured at 25 °C under shear rates ranging from 0.1 to 1000 s^−1^.

### 2.6. Dynamic Frequency Sweep

#### 2.6.1. Strain Sweep Test

Perform strain sweeps on RTW-1 samples at different concentrations at 25 °C to determine their linear viscoelastic region. Apply strains ranging from 0.1% to 100% to the samples while maintaining a frequency of 1 Hz to analyze their linear viscoelastic range (LVR).

#### 2.6.2. Frequency Scan Test

Using the linear viscoelastic region obtained from Test 2.6.1, measure the relationship between storage modulus (*G*’) and loss modulus (*G*″) for RTW-1 samples at different concentrations across frequencies from 0.1 to 10 Hz under constant strain.

### 2.7. Creep–Recovery Test

Conduct creep–recovery tests on RTW-1 solutions of varying concentrations at 25 °C within a scanning range of 0.01–100 Pa. Maintain a stress of 0.1 Pa during the creep scan for 150 s. Maintain a stress of 0 Pa during the recovery scan for 450 s.

### 2.8. Thixotropy Testing

The rheological properties of the RTW-1 solution were evaluated using a thixotropic hysteresis loop test. Under constant temperature conditions of 25 ± 0.1 °C, a linearly increasing shear rate (0.1–1000 s^−1^) was first applied, followed by a symmetrical decreasing scan (1000–0.1 s^−1^). The relationship between apparent viscosity (*η*) and shear rate (*γ*) was monitored in real time. The enclosed area of the hysteresis loop showed a positive correlation with thixotropic properties.

### 2.9. In Vitro Digestive Simulation Experiment of RTW-1

#### 2.9.1. In Vitro Salivary Digestive Simulation Test of RTW-1

##### Saliva Preparation

Simulated saliva: Weigh 1.195 g Na_2_HPO_4_, 0.095 g KH_2_PO_4_, 4 g NaCl, and 0.059 g α-amylase. Dissolve in deionized water, adjust to 500 mL volume, then adjust the pH to 6.75 using phosphate buffer solution. Reserve for later use.

##### In Vitro Simulated Saliva Digestion Test

Ten milliliters of a 2 mg/mL RTW-1 sample solution are prepared. Mix with 15 mL of the simulated saliva prepared as above. Incubate at 37 °C with shaking for 1 h. Samples were collected at time points 0, 0.25, 0.75, and 1 h. Each sample was boiled for 10 min to inactivate enzymes, halting the reaction for subsequent use.

#### 2.9.2. In Vitro Digestive Assay of RTW-1 in Simulated Gastric Fluid

##### Preparation of Gastric Juice

Preparation of simulated gastric electrolyte solution: Weigh 0.6 g NaHCO_3_, 3.1 g NaCl, 1.1 g KCl, and 0.15 g CaCl_2_·2H_2_O. Dissolve in 1 L of distilled water. Adjust the pH to 3.0 using hydrochloric acid.

Artificial gastric juice preparation: Weigh 35.4 mg pepsin, 37.5 mg lipase, 1.5 mL 1 mol/L CH_3_COONa solution (pH 5), and 150 mL gastric electrolyte solution. The actual pH of gastric fluid is influenced by various factors, including food intake. Post-ingestion, gastric pH may rise to approximately 3.5 due to food dilution. Adjust the final system pH to 3.0 using hydrochloric acid titration.

##### In Vitro Simulated Gastric Digestion Assay

Mix 10 mL of the saliva solution processed in step 2.9.1 with 15 mL of pre-prepared artificial gastric fluid. Incubate at 37 °C with continuous agitation to maintain the digestion process for 6 h. During this process, samples were collected at 0, 2, 4, and 6 h. Subsequently, the samples were boiled for 10 min to inactivate enzymes, thereby terminating the digestion reaction for subsequent use.

#### 2.9.3. In Vitro Digestive Assay of RTW-1 Simulating Intestinal Fluid

##### Preparation of Intestinal Fluid

Preparation of intestinal electrolyte solution: Contains 5.4 g/L NaCl, 0.65 g/L KCl, and 0.33 g/L CaCl_2_·2H_2_O.

Preparation of pancreatin solution: A 7% pancreatin solution was prepared, stirred thoroughly, centrifuged, and the supernatant was collected.

Preparation of intestinal fluid: Thoroughly mix 50 g of intestinal electrolyte solution, 50 g of pancreatic enzyme solution, and 100 g of 4% bile salt solution. Add 6.5 mg of trypsin and adjust the pH of the mixture to 7.

##### In Vitro Simulated Intestinal Digestion Test

Take 10 mL of the solution digested with gastric juice as described in 2.9.2. and mix it with 15 mL of the prepared intestinal fluid. Incubate the mixture at 37 °C with shaking for 6 h. During digestion, samples were collected at 0, 2, 4, and 6 h. Each sample was boiled for 10 min to inactivate the enzymes, terminate the reaction, and preserve it for subsequent use.

### 2.10. Immunological Activity of RTW-1

#### 2.10.1. Cell Culture

RAW264.7 cells were provided by the Innovation Team of Edible and Medicinal Mushrooms and Health Products, Tianjin University of Science and Technology, with catalog no. 0806. The cells were cultured in DMEM (SolarBio Co., Beijing, China) supplemented with 10% fetal bovine serum and incubated at 37 °C in a CO_2_ incubator.

#### 2.10.2. Effects on Cell Proliferation Activity

Transfer logarithmic growth phase cells to a 96-well plate (200 μL/well), adjusting the density appropriately. After cells achieved full confluence, the medium was replaced with RTW-1-containing culture medium (0, 10, 20, 40, 80, 160, and 320 μg/mL), again at 200 μL per well. Lipopolysaccharide (SolarBio Co., Beijing, China) (LPS, 1 μg/mL) served as the positive control. After 12 and 24 h of incubation, cell proliferation was measured using the CCK-8 assay kit (SolarBio Co., Beijing, China).

#### 2.10.3. Effects on Cell Phagocytic Activity

Transfer logarithmic growth phase cells to a 96-well plate (200 μL/well), adjusting density appropriately. Allow cells to fully adhere. Replace with culture medium containing RTW-1 (0, 10, 20, 40, 80, 160, and 320 μg/mL), with a final volume of 200 μL per well. Use LPS (1 μg/mL) as a positive control. After 24 h of incubation, wash away residual medium. A volume of 100 μL of 0.1% neutral red solution was added to each well, followed by incubation in the dark for 4 h. After the solution was aspirated and discarded, 200 μL of cell lysis buffer was added to each well. The plate was then incubated in the dark for 1 h, after which the OD value was measured at 540 nm.

#### 2.10.4. Study of Cellular NO Production

Transfer logarithmic growth phase cells to a 96-well plate (200 μL/well) at an appropriate density. The cells were allowed to adhere. Replace the medium with RTW-1-containing culture medium (0, 10, 20, 40, 80, 160, and 320 μg/mL), adding 100 μL per well. Use LPS (1 μg/mL) as the positive control and incubate for 24 h. Prepare NO standards at concentrations of 0, 1, 2, 5, 10, 20, 40, and 100 μM. Add Griess Reagent I and II from the NO detection kit (SolarBio Co., Beijing, China). Absorbance was measured, and the nitrite concentration was calculated from the standard curve.

#### 2.10.5. Effect on Cell Enzyme Activity

Logarithmically growing cells were seeded into 6-well plates at an appropriate density (2 mL/well) and were allowed to adhere completely. Replace the medium with RTW-1-containing culture medium (0, 10, 20, 40, 80, 160, and 320 μg/mL), again at 2 mL per well. Use LPS (1 μg/mL) as the positive control and incubate for 24 h. Gently disperse the cells in the flask, wash with PBS, and collect them for subsequent cell enzyme activity assays.

#### 2.10.6. Effects of Cellular Acid Phosphatase (ACP)

Resuspend the collected RAW264.7 cells in PBS, disrupt the cells using an ultrasonic device, and centrifuge for separation. Following the ACP assay kit (SolarBio Co., Beijing, China) protocol, ACP activity levels were measured in samples treated with different concentrations of RTW-1, as well as in blank controls and positive controls.

#### 2.10.7. Effects of Cellular Lysozyme (LZM)

The collected RAW264.7 cells were resuspended in PBS, disrupted using an ultrasonic device, and then centrifuged. Following the LZM assay kit (SolarBio Co., Beijing, China) protocol, LZM activity levels were measured in samples treated with different concentrations of RTW-1, as well as in blank controls and positive controls.

#### 2.10.8. Effects of Cellular Superoxide Dismutase (SOD)

The collected and preserved RAW264.7 cells were resuspended in PBS, disrupted using an ultrasonic device, and then centrifuged. Following the SOD assay kit (SolarBio Co., Beijing, China) protocol, SOD activity levels were measured in samples treated with RTW-1 at various concentrations, alongside blank controls and positive controls.

#### 2.10.9. Effects of Cell Morphology

Transfer logarithmic growth phase cells to 6-well plates (2 mL/well) at an appropriate density. Allow cells to fully adhere. Replace the medium with RTW-1-containing culture medium (0, 20, 40 μg/mL), again at 2 mL per well. Use LPS (1 μg/mL) as the positive control and incubate for 24 h. Wash away the medium with PBS. Proceed with subsequent AO staining and PAS staining.

##### Acridine Orange (AO) Staining

Cells were fixed with cell fixative prior to adding acetic acid. The fixative and acetic acid were then discarded. Cells were stained with AO solution (0.01%) for 20 min under dark conditions. Calcium chloride solution (0.1 mol/L) was subsequently added, followed by washing with PBS. Images were captured using an inverted fluorescence microscope.

##### Glycogen (PAS) Staining

Following the method of Valentine et al., add 100 μL of periodic acid solution, then transfer the sample to a humidity chamber in a dark environment for 10 min [31]. After the reaction is complete, immerse in distilled water and gently agitate for 5 min to complete the initial rinse. Next, add 100 μL Schiff’s reagent solution and incubate in a humidified box protected from light for 60 min. After staining, immerse the sample in distilled water and gently agitate for 5 min to thoroughly remove residual reagents. Perform rapid nuclear staining with 100 μL hematoxylin, followed by washing and microscopic examination.

### 2.11. Effects on mRNA Expression Levels of Immune Factors in Cells

#### 2.11.1. RNA Extraction from RAW264.7 Cells

RAW264.7 cells were cultured with RTW-1 polysaccharide solutions at concentrations of 0, 20, 40, 80, 160, and 320 μg/mL as experimental groups, with parallel controls. The blank control group was treated with complete medium, while the positive control group was treated with a 1 μg/mL LPS solution. After 24 h of culture, cells were harvested, and total RNA was extracted using the E.Z.N.A. Total RNA Kit I (SolarBio Co., Beijing, China).

#### 2.11.2. Primer Design

Primers were synthesized by Tianjin Geneview Biotechnology Co., Ltd. (Tianjin, China). For subsequent gene expression analysis, β-actin was used as the housekeeping gene. Polymerase chain reaction (PCR) was performed using cDNA as the template. The relevant primer sequences are shown in Table 1.

#### 2.11.3. qRT-PCR Detection of Immune Factor Gene Expression in RAW264.7 Cells

Reverse transcription was performed using a reverse transcription kit (Takara Bio Inc., San Jose, CA, USA) to convert RNA into cDNA. The total reaction volume was 20 μL, composed as follows: 2 μL cDNA, 7.2 μL RNase-free H_2_O, 0.4 μL forward primer, 0.4 μL reverse primer, and 10 μL TB Green Premix Ex Taq II. The reaction protocol was as follows: the temperature was maintained at 95 °C for 30 s, followed by 40 cycles of PCR amplification at 95 °C for 5 s, 60 °C for 20 s, and 72 °C for 30 s (Detailed procedures are provided in the Supplementary Material Tables S1–S4).

## 3. Results and Discussion

### 3.1. Preparation of RTW-1

**Figure 1:**
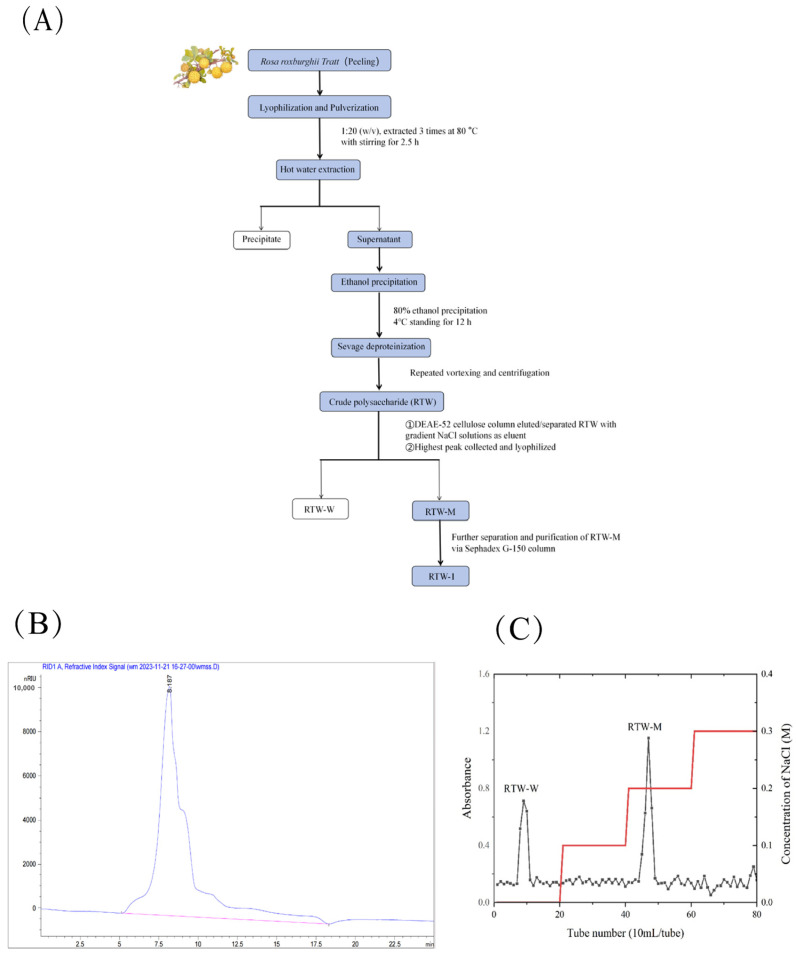
Flowchart for the preparation of RTW-1 (**A**). RTW phase diagram for raw sugar liquors (**B**). DEAE-52 cellulose purification elution curve of RTW (**C**).

The crude polysaccharide obtained from *Rosa roxburghii* Tratt powder after water extraction, ethanol precipitation, protein removal via the Sevag method, dialysis, and lyophilization was designated as RTW. Analysis by high-performance liquid chromatography was performed, as shown in Figure 1B. Subsequent separation and purification via a DEAE-52 cellulose column yielded two fractions, designated RTW-W and RTW-M, as shown in Figure 1C. A larger peak area and higher yield were exhibited by the NaCl-eluted fraction compared to RTW-W. Therefore, RTW-M was selected as the primary component for *Rosa roxburghii* Tratt polysaccharide research, and it was collected via dialysis and freeze-dried for subsequent use. The RTW-M fraction collected from Figure 1B was purified using Sephadex G-150. The apex and symmetrical points of the ascending and descending slopes were collected, freeze-dried, and designated as RTW-1. This fraction contained a sugar content of 91.32%. The corresponding standard curve is provided in Supplementary Figure S1. The fraction was reserved for subsequent experiments.

### 3.2. Determination of Molecular Weight

**Figure 2:**
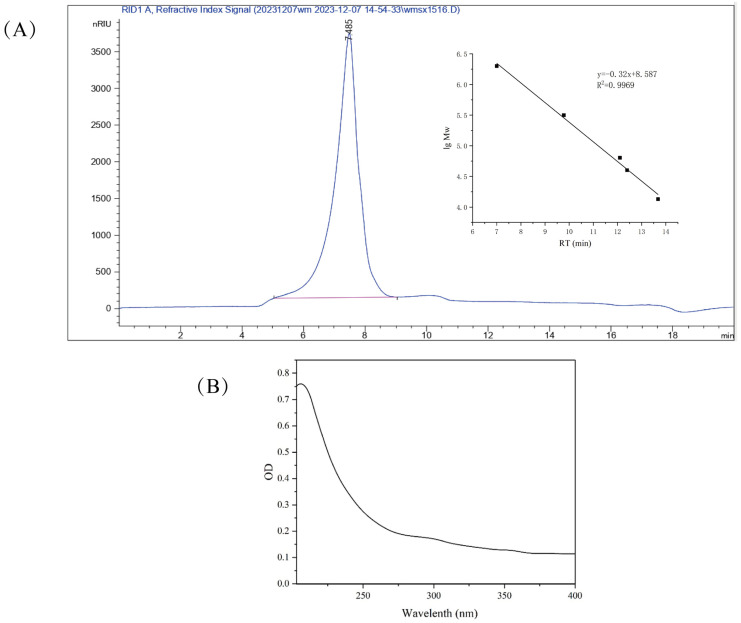
RTW-1 high performance liquid chromatogram and molecular weight standard curve of dextran standard (**A**). RTW-1 UV–Vis scanning chart (**B**).

As shown in Figure 2A, the elution times were concentrated between 5 and 10 min, with a molecular weight distribution ranging from 1.35 × 10^3^ kDa to 2.72 × 10^3^ kDa. The distribution was dominated by a single peak, indicating that the peak at 7.34 min represented the major component of RTW. Its molecular weight was determined to be 2.16 × 10^3^ kDa, as shown in Figure 2A. The high molecular weight of RTW-1 typically resulted in increased solution viscosity. Recently, an acidic heteropolysaccharide (BSP) isolated from *Brasenia schreberi*, with a molecular weight of 2.47 × 10^4^ Da, was reported to exhibit significant shear-thinning behavior attributable to chain entanglement and network formation [32]. This behavior provided a structural basis for the pseudoplastic and weak-gel properties observed in subsequent rheological analyses.

### 3.3. UV Full-Wavelength Scan Analysis of Polysaccharides

A full-wavelength scan of RTW-1 was performed, and the results are shown in Figure 2B. No absorption peaks were exhibited by RTW-1 at 260 nm or 280 nm, indicating the absence of nucleic acids and proteins. Potential interference from proteins and nucleic acids in subsequent immunomodulatory studies was avoided. The high purity of RTW-1 (91.32% sugar content) ensured that the observed biological activity could be unequivocally assigned to the polysaccharide itself and not to co-extracted impurities.

### 3.4. Uronic Acid Content Determination

The uronic acid content of RTW-1 was determined to be 55.21%, indicating that it is an acidic polysaccharide (The standard curve is available in Supplementary Figure S2). The high uronic acid content of RTW-1 provided a basis for its structural analysis and immunomodulatory evaluation. In particular, the presence of galacturonic acid effectively enhances the expression of immune activity [33].

### 3.5. Esterification Degree Analysis

Calculations indicated that RTW-1 exhibited an esterification degree of 58.30%, demonstrating a high level of esterification. A recent study on a rhamnogalacturonan-I fraction isolated from carrot reported that cytokine levels were largely governed by molecular weight and degree of esterification. The fraction with the highest molecular weight (100 kDa) exhibited the strongest immunomodulatory activity, whereas a marked reduction in activity was observed following saponification (removal of ester groups) [34]. These findings suggested that ester groups not only served as structural features but were also actively involved in receptor recognition and immune signaling. This provided a basis for further investigations into the activity of RTW-1.

### 3.6. Scanning Electron Microscopy Analysis

**Figure 3:**
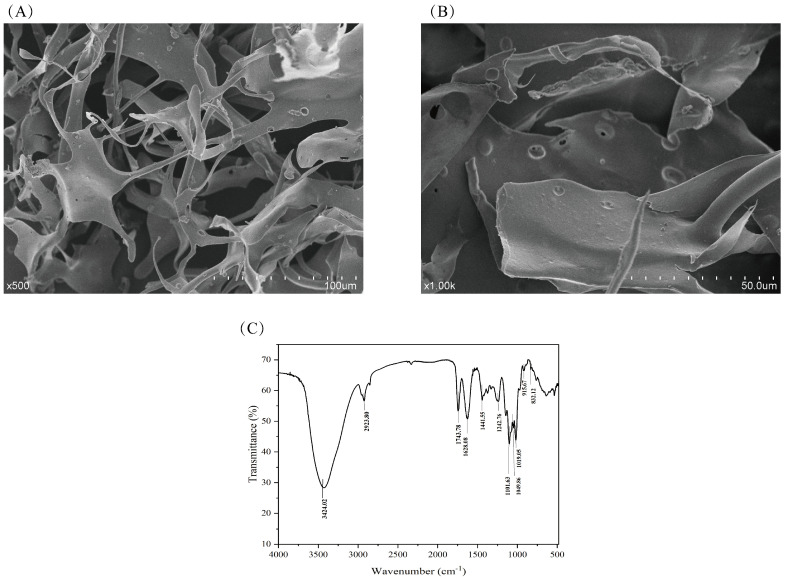
SEM image of RTW-1 ((**A**): 500×; (**B**): 1000×). RTW-1 FT-IR spectrogram (**C**).

Scanning electron microscopy (SEM) revealed the specific morphological characteristics of the polysaccharide, which are directly related to its relative molecular mass and chemical composition. As shown in Figure 3A,B, at magnifications of 500× and 1000×, respectively, a plate-like structure was exhibited by the SEM images of RTW-1. At 500× magnification, aggregated and layered features were predominantly displayed, indicating strong intermolecular forces within RTW-1. Combined with the aforementioned molecular weight determination, this characteristic may also be attributed to its high molecular weight [35]. Not only intricate intertwining and aggregation but also substantial gaps and a loosely arranged interior were exhibited on the surface of RTW-1. This structural feature was considered conducive to efficient cross-linking with other compounds.

### 3.7. Analysis of Fourier Transform Infrared Spectroscopy (FT-IR) Results

The molecular structure of RTW-1 was analyzed using Fourier Transform Infrared Spectroscopy (FT-IR), and the results are shown in Figure 3C. Characteristic absorption peaks were observed at 3424.02 cm^−1^ and 2923.80 cm^−1^, corresponding to O-H stretching and C-H stretching vibrations [36], respectively. This indicated the presence of functional groups such as hydroxyl, alkyl, or methyl groups within the RTW-1 molecule. The characteristic absorption peak at 1743.78 cm^−1^ was attributed to the C=O stretching vibration in an acetyl group or carboxylate ester, a signature peak for acidic sugars, confirming RTW-1 as an acidic polysaccharide [37]. The absorption peak of bound water was identified at 1628.08 cm^−1^, and the stretching vibration peak of the carboxyl C-O bond was observed at 1441.55 cm^−1^. The presence of a carboxylate group in RTW-1 was primarily confirmed by the characteristic absorption peak of C-O-C at 1242.76 cm^−1^ in the infrared spectrum. As shown in Figure 3C, the absorption peaks at 1101.63 cm^−1^, 1049.86 cm^−1^, and 1019.05 cm^−1^ indicated the presence of a pyranose ring in RTW-1 [38,39]. The absorption peak at 915 cm^−1^ was identified as characteristic of β-glycosidic bonds, and the peak at 832.12 cm^−1^ was assigned to α-glycosidic bonds. This further confirmed the distribution of glycosidic bond types within the RTW-1 molecule.

### 3.8. Congo Red Experimental Analysis

**Figure 4:**
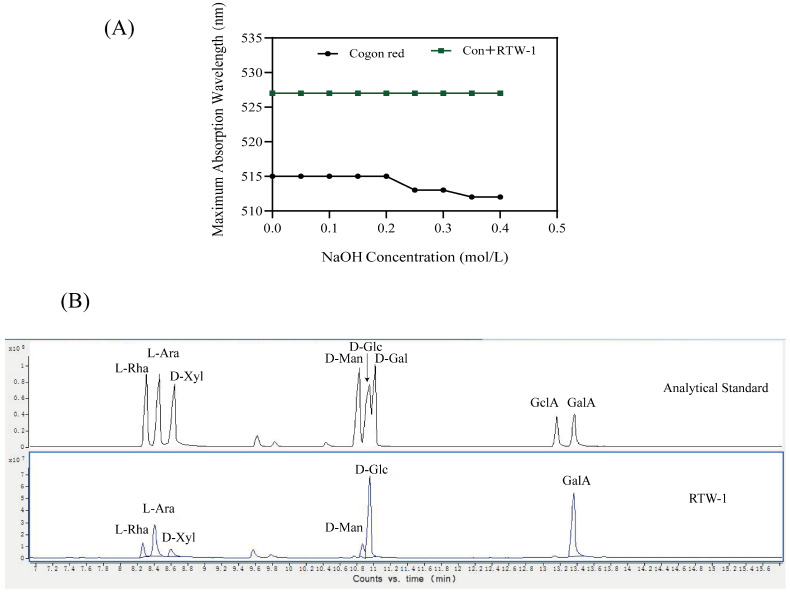
The experimental wavelength of Congo Red (**A**). Gas chromatogram of monosaccharide standard and RTW-1 monosaccharide composition (**B**).

As presented in Figure 4A, no observable change was exhibited by the Congo Red solution in the control group upon increasing NaOH concentration. In contrast, a gradual decrease in the peak absorbance value was observed in the experimental group with increasing NaOH concentration, although the change was not significant. Therefore, RTW-1 does not contain a helical structure. This result was consistent with findings reported for a low-molecular-weight polysaccharide (CSP1a, 15.7 kDa) isolated from artificially cultivated *Cordyceps militaris* [28]. In the Congo Red assay, this polysaccharide displayed a reticular porous chain conformation rather than a triple helix. Nevertheless, it still exhibited significant immunomodulatory activity and enhanced bioavailability.

### 3.9. Monosaccharide Composition Analysis

Through degradation and processing of polysaccharides, the monosaccharide composition of the polysaccharide sample RTW-1 was systematically analyzed using gas chromatography. As shown in Figure 4B, the chromatogram of the reference standards (upper) was compared with that of the RTW-1 sample (lower). The peaks in the upper reference chromatogram correspond to L-Rha, L-Ara, D-Xyl, D-Man, D-Glc, D-Gal, GlcA, and GalA. By comparing the characteristic peaks of the two spectra, RTW-1 was determined to contain six monosaccharide components: rhamnose, arabinose, xylose, mannose, glucose, and galacturonic acid. Their relative molar proportions, as determined by integration, were 0.15:0.48:0.14:0.19:1.00:0.74. This indicated that RTW-1 was predominantly composed of glucose, followed by galacturonic acid and arabinose, whereas the relative contents of rhamnose, xylose, and mannose decreased successively. The analysis of monosaccharide composition provided a foundation for subsequent.

A review on the structure–activity relationship of *Lycium barbarum* polysaccharides further highlights that arabinose and galactose contents serve as key structural determinants of immunomodulatory activity [40]. Moreover, arabinogalactan-like motifs are recognized as important recognition elements for immune cell activation. A homogeneous polysaccharide (SP4002501) was isolated from *Saposhnikoviae Radix* [41]. Its monosaccharide composition consisted of rhamnose, galacturonic acid, galactose, and arabinose at a molar ratio of 3.7:86.6:2.7:7.1. Galacturonic acid was the predominant component. This polysaccharide exhibited immunomodulatory activity by promoting macrophage proliferation and phagocytosis. Monosaccharide composition analysis provided an essential structural basis for subsequent methylation and rheological studies. Furthermore, it established a foundation for further elucidation of the structure–activity relationship of RTW-1.

### 3.10. Methylation Analysis

Methylation analysis is crucial for determining glycosidic bond linkages [42]. Sample RTW-1 underwent methylation treatment, followed by degradation and reduction, and then was analyzed via GC-MS. The test results were compared with data in the CCRC spectral database for partially methylated polyhydroxy sugar alcohol acetate (PMAA). The results are shown in Table 2. Six methylated sugar residues were identified in RTW-1: 6-O-Me1-Man*p*, 2,3-O-Me2-Ara*p*, 2,3,6-O-Me3-Gal*p*, 2,3, 4-O-Me3-Rha*p*, 2,3,6-O-Me3-Glc*p*, and 1,2,3,4,5-O-Ac5-Xyl*p*. Their relative molar ratios were 0.04:0.16:0.74:0.09:1.00:0.11, respectively. 2,3,6-O-Me3-Gal*p* is the product of the reduction of the carboxyl group of GalA to a hydroxyl group during the methylation of galacturonic acid. GalA is converted to Gal. Since Gal was not detected in the monosaccharide composition analysis of RTW-1, it was considered that all methylated Gal is derived from the reduction of GalA. The GalA linkage pattern was determined to be (1→4) [43].

The proportions of individual sugar residues in the methylation analysis largely correspond with the monosaccharide composition analysis. In summary, RTW-1 was determined to be primarily composed of a highly branched macromolecular polysaccharide consisting of →2,3,4)-Manp-(1→, →4)-Arap-(1→, →4)-GalAp-(1→, T-Rhap, →4)-Glcp-(1→,→2,3,4)-Xylp- (1→). The high degree of branching was found to result in poor fluidity and high viscosity at elevated concentrations. Reduced steric hindrance facilitated chain entanglement under these conditions. Consistent with prior rheological findings, Zhao et al. [30] observed similarly highly branched polysaccharides extracted from passion fruit peel via high-shear, low-temperature processing. Mirroring this study, the solution exhibited pseudoplastic fluid behavior, with viscosity decreasing as shear rate increased.

Zhang et al. reported two pectic polysaccharides, RTFP1-2 and RTFP2, with molecular weights of 112 kDa and 506 kDa, respectively [44]. Both were rich in galacturonic acid (53–65%) and contained homogalacturonan (HG) and rhamnogalacturonan I (RG-I) domains. Chen et al. isolated a heteropolysaccharide fraction, designated RTFPs, which was composed of two molecular-weight components (187 kDa and 4.75 kDa). The major monosaccharides identified in this fraction were glucose (38.93%), arabinose (20.66%), galactose (20.58%), and galacturonic acid (10.94%) [19]. This fraction showed immunomodulatory activity in RAW264.7 macrophages. Liu et al. further isolated a low-molecular-weight polysaccharide, designated RRTP80-1, with a molecular weight of 8.65 kDa. This polysaccharide was composed exclusively of arabinose, glucose, and galactose and contained no uronic acids. Its glycosidic linkages included →5)-Ara*f*-(1→ and →6)-Glc*p*-(1→ residues [16].

In comparison, RTW-1 shows several notable structural features. First, its molecular weight (2.16 × 10^3^ kDa) was substantially higher than those of previously reported *Rosa roxburghii* polysaccharides (8.65–506 kDa). Second, although RTW-1 was mainly composed of glucose, arabinose, and galacturonic acid, its glycosidic linkage pattern was found to include (→2,3,4)-Man*p*-(1→ and (→2,3,4)-Xyl*p*-(1→. These structural differences, together with the high viscosity and shear-thinning rheological behavior of RTW-1, indicated that RTW-1 was distinct from previously reported *Rosa roxburghii* polysaccharides. Further investigation of its structure–function relationship may therefore be warranted.

### 3.11. Rheological Results Analysis

#### 3.11.1. Steady-State Rheological Testing Analysis

**Figure 5:**
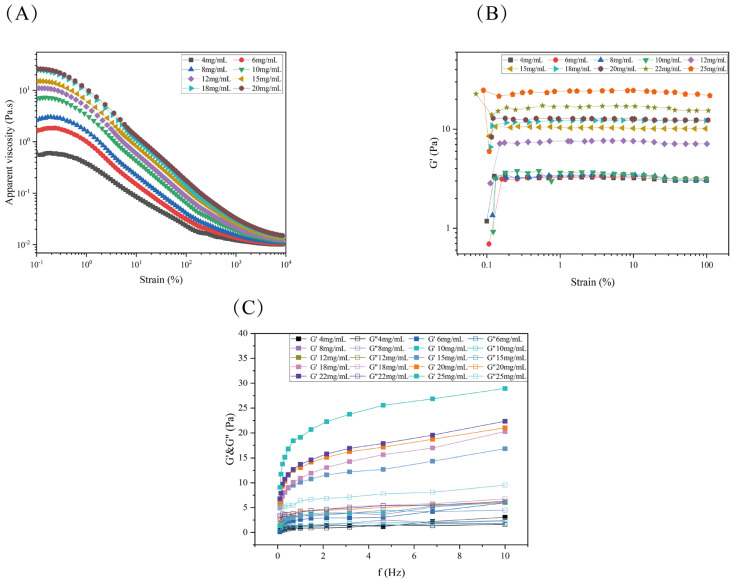
Dependence of steady-state shear viscosity of RTW-1 with different concentrations on shear rate (**A**). Different concentrations of RTW−1 strain scanning (**B**). RTW−1 Dynamic Frequency Scan (**C**).

The apparent viscosity (η) of RTW-1 at different concentrations was influenced by shear rates (γ) ranging from 0.1 s^−1^ to 1000 s^−1^. As shown in Figure 5A, RTW-1 exhibits high apparent viscosity at low shear rates. At all concentrations, apparent viscosity was observed to decrease gradually with increasing shear rate. This indicates that molecular chain interactions are progressively disrupted as shear rate increases, leading to weakened intermolecular forces [45]. Under shear stress, free macromolecular chains in the solution transition from a disordered, freely flowing state to an ordered state flowing along the shear direction. As a result, intermolecular spacing was increased, interactions such as hydrogen bonds were weakened, and rearrangement between molecules was induced. Consequently, pronounced shear-thinning behavior was observed, which is characteristic of pseudoplastic fluids. The observed pseudoplastic behavior is consistent with that reported for other polysaccharides. For instance, Tremella fuciformis polysaccharide was found to exhibit shear-thinning flow in aqueous solutions due to the shear-induced alignment of entangled worm-like chain networks [46]. Similarly, Cyperus esculentus polysaccharide was found to enhance resistance to shear thinning in starch–polysaccharide complexes [47].

#### 3.11.2. Dynamic Frequency Sweep Analysis

To characterize the viscoelastic properties of the RTW-1 solution, the linear viscoelastic region was determined via strain scanning. As shown in Figure 5B, when the applied oscillatory strain reached 1%, the storage modulus (*G*′) of the sample system stabilized, indicating that the network structure of RTW-1 did not undergo irreversible damage at this strain level. Therefore, 1% was selected as the constant strain condition for frequency scanning tests.

The storage modulus (*G*′) and loss modulus (*G*″) characterize the elastic and viscous properties of the solution. As shown in Figure 5C, both G′ and G″ values were observed to exhibit a positive correlation with frequency, with G′ generally exceeding G″. Across all tested frequencies, G′ was found to be greater than G″ for RTW-1 solutions at different concentrations. This demonstrates weak gel rheological properties, indicating a gel network structure with pronounced elastic characteristics. This behavior is typically correlated with strong intermolecular interactions, such as hydrogen bonds and hydrophobic interactions.

Both G′ and G″ were observed to increase with rising frequency. This indicates that molecular chains are more readily arranged in an ordered manner at high frequencies, thereby forming transient network structures that enhance elastic behavior. The frequency dependence demonstrates that the gel network structure of RTW-1 solutions exhibits greater elasticity at higher polysaccharide concentrations. Similar weak gel behavior with G′ exceeding G″ has been reported for other polysaccharide systems, including blueberry leaf polysaccharide–gelatin composites [48] and xanthan gum–*Gleditsia sinensis* polysaccharide blends [49].

#### 3.11.3. Creep–Recovery Test Analysis

**Figure 6:**
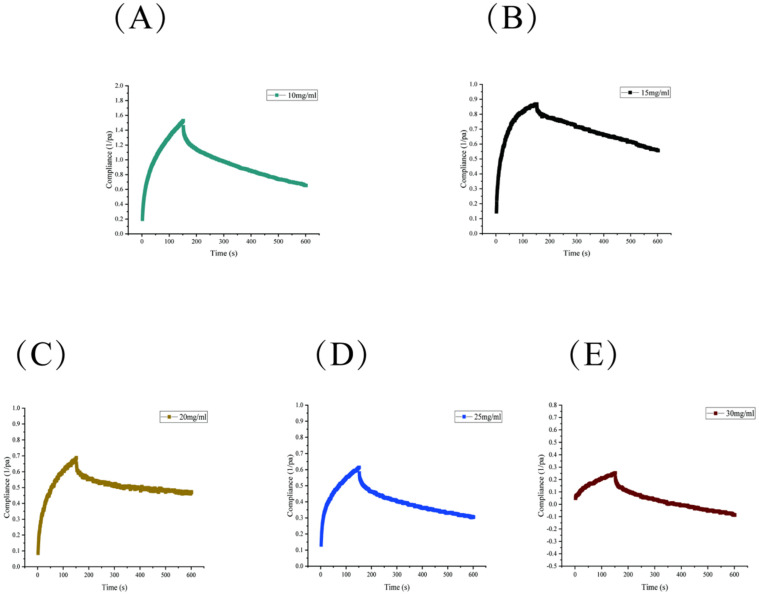
Creep–recovery curves of RTW−1 at different concentrations. (**A**) 10 mg/mL RTW−1; (**B**) 15 mg/mL RTW−1; (**C**) 20 mg/mL RTW−1; (**D**) 25 mg/mL RTW−1; (**E**) 30 mg/mL RTW−1.

Creep analysis involves rapidly deforming samples under applied stress to evaluate their transient viscoelastic properties. As shown in Figure 6A–E, during the 0–150 s creep scan, the deformation time was observed to increase when sample RTW-1 was subjected to constant stress.

As the concentration was increased from 10 to 30 mg/mL, the total deformation of the solution was found to decrease gradually, indicating enhanced creep resistance. Upon stress removal, the deformation of RTW-1 was observed to decrease over time until reaching a steady state. This behavior indicates viscous flow characteristics and irreversible deformation. As concentration increased, total deformation decreased while hydrogen bonding and intermolecular interactions among RTW-1 molecules were strengthened, progressively enhancing the rigidity of the gel network structure. The observed concentration-dependent enhancement in creep resistance is in line with findings from other polysaccharide gel systems. Yang et al. [50] demonstrated that flaxseed gum/konjac glucomannan composite gels exhibited increased creep resistance with higher polysaccharide content, attributable to the formation of a denser three-dimensional network structure. Similarly, Wang et al. [51] reported that salecan/konjac glucomannan biogels showed distinct creep behaviors with maximum creep compliance and high recovery rates dependent on gel composition, paralleling the concentration-dependent network reinforcement observed for RTW-1.

#### 3.11.4. Thixotropy Test Analysis

**Figure 7:**
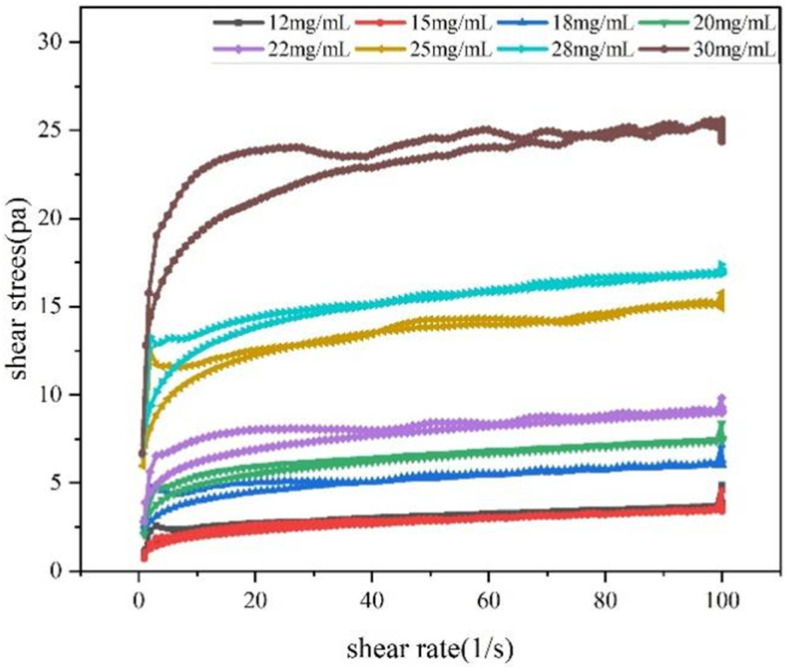
The thixotropy of RTW−1 with various concentrations.

Thixotropy refers to a sample solution’s ability to regain viscosity after undergoing external shear. The disruption of its internal structure manifests as varying hysteresis loop areas, which form distinct thixotropic loops [47] that quantify the thixotropic strength of the fluid. As shown in Figure 7, a positive correlation was observed between the thixotropy of the RTW-1 solution and polysaccharide concentration. The thixotropic loop areas at increasing concentrations were determined to be 7.10, 7.30, 11.40, 23.60, 33.74, 45.91, 53.72, and 143.80 Pa/s, respectively. When an external force was applied, a significant change in system viscosity was observed. However, upon removal of the external force, a considerable time was required for the system to return to its original unforced state. Meng et al. [52] reported that exopolysaccharide from Schizophyllum commune exhibited concentration-dependent thixotropy. Cheng et al. [53] demonstrated that the thixotropic loop area of Clitocybe squamulosa polysaccharide increased with mass fraction. These results support that the concentration-dependent enhancement of thixotropy observed in RTW-1 is characteristic of high-molecular-weight polysaccharides.

#### 3.11.5. Summary of Rheological Experiments

Based on the physicochemical properties of RTW-1, it was determined to be an acidic polysaccharide with a high esterification degree and poor solubility. Therefore, rheological characterization was employed to systematically illustrate the rheological properties of the RTW-1 solution, as presented in Figure 5, Figure 6 and Figure 7. In steady-state shear tests, shear-thinning behavior was observed, with viscosity differences across concentrations diminishing gradually as shear rate increased, indicating pseudoplastic fluid behavior. Dynamic viscoelastic testing revealed a critical linear viscoelastic region threshold at 1% strain during strain scanning. Frequency scanning at this strain showed *G*′ consistently exceeding *G*″, confirming the RTW-1 solution exhibits weak gel properties. This elastic-dominant behavior was attributed to transient network structures formed by intermolecular hydrogen bonds and hydrophobic interactions. Creep analysis indicated that creep resistance was significantly enhanced with increasing concentration, which was ascribed to strengthened molecular chain cross-linking networks at higher concentrations. Thixotropy testing revealed a positive correlation between thixotropic loop area and concentration. The loop area at 30 mg/mL (143.8 Pa/s) was found to be approximately 20-fold greater than that at 12 mg/mL (7.1 Pa/s), confirming that higher-concentration systems exhibit greater structural remodeling capacity. The concentration-dependent rheological behavior observed for RTW-1 is in general agreement with that reported for other polysaccharides. Abdin et al. [54] reported that corn silk polysaccharide exhibited shear-thinning behavior and enhanced elastic modulus with increasing concentration, while Hu et al. [55] demonstrated that lentinan showed obvious viscoelasticity and thixotropic behavior with significant concentration dependence. These findings support that the rheological responses of RTW-1 are consistent with the typical behavior of high-molecular-weight polysaccharides.

### 3.12. In Vitro Simulated Digestion Test of RTW-1

Research has indicated that polysaccharides exhibit a tendency to form aggregates in solution. This aggregation occurs due to the breaking of glycosidic bonds or the disruption of existing aggregates [56]. Changes in reducing sugar content provide an accurate indicator of this process. The breaking of glycosidic bonds leads to a significant increase in reducing sugar content. Therefore, changes in *Rosa roxburghii* Tratt polysaccharide during simulated digestion processes were specifically analyzed. These processes involved saliva, gastric juice, and intestinal fluid in vivo, and the analysis was performed by measuring changes in total sugar content [57].

As shown in Table 3, the undigested original reducing sugar content was 0.0395 ± 0.0100 mg/mL. Throughout the entire saliva digestion process, the reducing sugar content was observed to remain stable between 0.0409 ± 0.0100 mg/mL and 0.0421 ± 0.0100 mg/mL, with no significant variation detected. This stability was attributed to the mild oral environment, the specificity of salivary amylase, and the relatively short digestion time. This finding was consistent with the in vitro oral simulated digestion test results reported for *Zanthoxylum bungeanum maxim pericarp* polysaccharide by Liu et al. [58] and bitter melon polysaccharides studied by Zhu [59]. Upon transition from saliva to gastric fluid, an increase in reducing sugar levels to 0.0447 ± 0.0100 mg/mL was observed. This rise was likely attributed to alterations in digestive fluid composition and polysaccharide concentration. After gastric digestion, the reducing sugar content of RTW-1 was found to change from 0.0447 ± 0.0100 mg/mL to 0.0471 ± 0.0100 mg/mL. This variation was negligible, and the content remained relatively stable. After 6 h of simulated intestinal digestion, the reducing sugar content was determined to be 0.0537 ± 0.0100 mg/mL. Compared with the original reducing sugar content, the reducing sugar content of RTW-1 was concluded to have remained essentially stable after simulated digestion. No degradation of the polysaccharide sample RTW-1 was observed during simulated digestion with saliva, gastric juice, or intestinal fluid.

### 3.13. Immunological Activity Study of RTW-1

#### 3.13.1. Effects of RTW-1 on RAW264.7 Cell Proliferation

**Figure 8:**
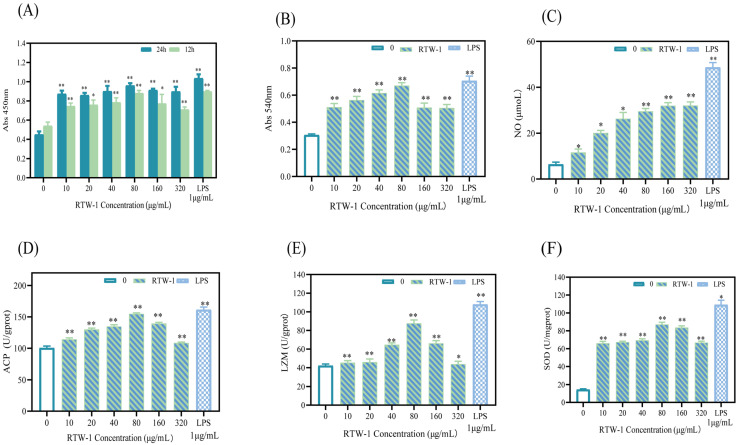
Effects of RTW-1 concentration and time on the proliferation activity of RAW264.7 cells (**A**). The effect of RTW-1 concentration on the phagocytic activity of RAW264.7 cells (**B**). Effect of RTW-1 concentration on NO release in RAW264.7 cells (**C**). The effect of RTW-1 concentration on ACP activity in RAW264.7 cells (**D**). The effect of RTW-1 concentration on LZM activity in RAW264.7 cells (**E**). The effect of RTW-1 concentration on SOD activity in RAW264.7 cells (**F**) (* *p* < 0.05; ** *p* < 0.01; compared with the blank group, the difference was statistically significant).

The CCK-8 assay was employed to evaluate the impact of RTW-1 on the proliferation activity of RAW264.7 cells treated at different time points. RAW264.7 cells were cultured with different concentrations of RTW-1 (0, 10, 20, 40, 80, 160, 320 μg/mL) and positive control LPS (1 μg/mL) for 12 h and 24 h. The results are shown in Figure 8A. Under both 12 h and 24 h culture conditions, RTW-1 enhanced the survival rate of RAW264.7 macrophages within a certain concentration range. Within this range, the polysaccharide RTW-1 exhibited no cytotoxicity toward RAW264.7 cells. At excessively high concentrations, cell density was observed to decrease. This was likely due to increased medium osmolarity, which inhibited proliferation during expansion. Overall, the 12 h treatment showed lower efficacy than the 24 h treatment. Consequently, 24 h was selected as the optimal exposure time, and concentrations ranging from 10 to 320 μg/mL were used for subsequent immunological activity studies.

In studies investigating the immunomodulatory activity of polysaccharides, the CCK-8 assay typically serves two primary functions. First, it verifies that the polysaccharide sample lacks cytotoxicity toward macrophages, thereby excluding the possibility that any observed immune enhancement results from toxic stress. Second, it defines a safe concentration range for subsequent immune functional assays [60]. This ensures that the detected immunomodulatory activity reflects the normal physiological state of the cells. In recent years, the CCK-8 assay has become an indispensable tool for basic characterization in polysaccharide-macrophage research, providing a reliable preliminary foundation for further mechanistic studies.

#### 3.13.2. Effects of RTW-1 on Phagocytic Activity in RAW264.7 Cells

The phagocytic activity of RAW264.7 macrophages effectively defends against pathogen invasion. Neutral red can be phagocytosed by cells. By measuring the internalized volume of neutral red dye in RAW264.7 macrophages, the phagocytic activity of macrophages under the influence of RTW-1 can be determined. Compared with the blank control group, phagocytic activity was increased by 44%, 58%, 71%, 88%, 40%, and 37% following treatment with RTW-1 at concentrations of 10, 20, 40, 80, 160, and 320 μg/mL, respectively. Thus, RTW-1 significantly enhanced the phagocytic activity of RAW264.7 cells in the concentration range of 10–80 μg/mL, with the maximum effect observed at 80 μg/mL, as shown in Figure 8B. This indicated that macrophages were effectively activated by RTW-1, thereby fully exerting its role in cellular immune regulation.

This finding is consistent with a recent study on mango seed kernel polysaccharide (MSKP), which was shown to enhance immune responses in RAW264.7 mouse macrophages by stimulating nitric oxide production, acid phosphatase activity, and phagocytosis [34]. The phagocytosis-enhancing effect of polysaccharides is typically mediated by pattern recognition receptors (PRRs) on the macrophage surface.

#### 3.13.3. Study on the Effect of RTW-1 on NO Production in RAW264.7 Cells

Phagocytosis in macrophages is closely linked to NO production. NO plays a crucial role in promoting cellular immune regulatory activity as a key effector molecule. When the body undergoes pathological stimulation, activated macrophages release the immunoregulatory cytokine NO to kill tumor cells [61]. Compared with the blank control group, NO release was increased by 26%, 125%, 237%, 304%, 327%, and 338% following treatment with RTW-1 at concentrations of 10, 20, 40, 80, 160, and 320 μg/mL, respectively. These findings demonstrated that NO release was promoted by RTW-1, as shown in Figure 8C.

#### 3.13.4. Effects of RTW-1 on Enzyme Activity in RAW264.7 Cells

##### Effects of RTW-1 on Acid Phosphatase (ACP) Activity in RAW264.7 Cells

ACP plays a crucial role in innate immune responses and exhibits high sensitivity to bacteria. Therefore, fluctuations in ACP levels directly correlate with an individual’s immune status and serve as a biomarker for assessing overall health. Compared with the blank control group, the measured activity was increased by 18%, 24%, 29%, 53%, 33%, and 11% following treatment with RTW-1 at concentrations of 10, 20, 40, 80, 160, and 320 μg/mL, respectively, as shown in Figure 8D. RAW264.7 macrophages were activated by RTW-1, and acid phosphatase (ACP) activity was enhanced. Maximum ACP activity was observed at 80 μg/mL. At higher concentrations, ACP activity declined but remained above the blank control level, possibly due to negative feedback regulation. Abdin et al. reported similar results, showing that mango seed kernel polysaccharide significantly promoted ACP activity in a dose-dependent manner. These findings further confirmed the link between ACP activity and immune activation [34].

##### Effects of RTW-1 on Lysozyme (LZM) in RAW264.7 Cells

LZM plays an indispensable role in immune defense and tissue repair processes. Compared with the blank control group, treatment with RTW-1 at concentrations of 10, 20, 40, 80, 160, and 320 μg/mL resulted in increases of 7.5%, 12.5%, 57.5%, 126%, 75%, and 5%, respectively, as shown in Figure 8E. RAW264.7 macrophages were influenced by RTW-1 treatment, and lysozyme (LZM) levels were observed to initially increase and then decrease. As with other enzyme activities, LZM activity reached its maximum at approximately 80 μg/mL of RTW-1. This confirmed that RAW264.7 macrophages were activated by RTW-1 and an immune response was triggered, thereby enhancing lysozyme activity. Further increases in the RTW-1 concentration led to a decline in lysozyme activity. This suggested that excessive RTW-1 may over-stimulate the cells. Consequently, counter-regulatory inhibition of lysozyme activity may occur. This concentration-dependent effect highlighted the importance of precise RTW-1 concentration control in optimizing macrophage activation during immune regulation. An exopolysaccharide isolated from the mycelium of *Cordyceps cicadae* was reported to significantly increase lysozyme, acid phosphatase, and superoxide dismutase levels in RAW264.7 cells, and to promote NO production [62], thereby effectively regulating macrophage immune activity. Therefore, lysozyme activity not only reflected the direct bactericidal capacity of macrophages but also indicated their finely tuned ability to modulate inflammatory responses and maintain immune homeostasis.

##### Effects of RTW-1 on Superoxide Dismutase (SOD) Activity in RAW264.7 Cells

Superoxide dismutase (SOD), a core component of the endogenous antioxidant defense system, exerts cellular protective effects by specifically scavenging superoxide anion radicals. Its activity level serves as a crucial immunological indicator for assessing the body’s antioxidant capacity. Compared with the blank control group, treatment with RTW-1 at concentrations of 10, 20, 40, 80, 160, and 320 μg/mL increased macrophage enzyme activity by 3.8%, 4.0%, 4.1%, 5.5%, 5.1%, and 4.2%, respectively. As shown in Figure 8F, within the concentration range of 10–80 μg/mL, the enzyme activity gradually increased and reached its maximum at approximately 80 μg/mL. This enhancement was attributed to the action of RTW-1 on macrophages in scavenging free radicals, thereby contributing to resistance against bacterial invasion. In the concentration range of 80–320 μg/mL, SOD activity remained significantly higher than that in the control group, although a decreasing trend was observed. This decline may be explained by a pro-oxidative effect induced by the high concentration of the polysaccharide. This effect caused excessive accumulation of reactive oxygen species (ROS) beyond the scavenging capacity of SOD. Consequently, oxidative damage occurred, and antioxidant capacity was reduced. These findings indicated that RTW-1 had the potential to optimize the antioxidant status of macrophages. A polysaccharide (FHVP-2) was isolated from *Ficus hirta* Vahl. In RAW264.7 cells, it effectively protected against H_2_O_2_-induced oxidative stress injury [63]. It also exhibited significant immunomodulatory activity. Another polysaccharide, acetylated *Morchella* polysaccharide (AEMP), was studied in a DSS-induced ulcerative colitis mouse model [64]. It significantly increased the activities of antioxidant enzymes, including SOD, CAT, and GSH-Px. These effects demonstrated enhanced antioxidant and immunomodulatory functions in vivo. Together, these findings indicated that the upregulation of SOD activity in macrophages by polysaccharides was an important mechanism. This mechanism enhanced cellular antioxidant capacity and contributed to the maintenance of redox homeostasis during immune activation.

#### 3.13.5. Effects of RTW-1 on RAW264.7 Cell Morphology

##### Acridine Orange (AO) Staining

Acridine Orange (AO) binds to intracellular nucleic acids, generating a specific fluorescent signal. During cell staining, normal cell nuclei exhibit uniform green or yellow-green fluorescence, whereas necrotic cells show reduced fluorescence intensity or may fail to fluoresce altogether.

**Figure 9:**
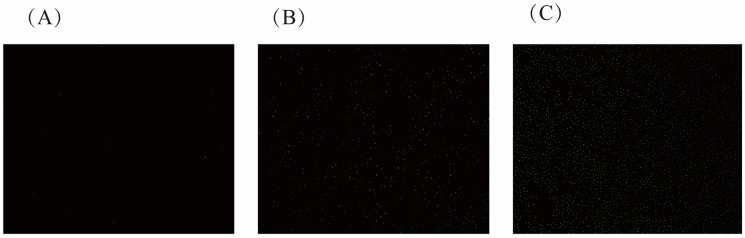
AO staining of RAW264.7 cells treated by RTW-1; (**A**) control group; (**B**) 20 μg/mL RTW-1; (**C**) 80 μg/mL RTW-1.

As shown in Figure 9A–C, at an RTW-1 concentration of 0 μg/mL, RAW264.7 cells exhibited small cell volume, reduced cell number, and weak fluorescence signals. This indicated that without RTW-1 stimulation, the cells’ growth and proliferation capacity was limited. Upon increasing the RTW-1 concentration, a significant increase in the number of RAW264.7 cells was observed. This was accompanied by brighter green fluorescence in the nuclear region, enlarged cell volume, accumulation phenomena, and a corresponding enhancement in the green fluorescence signal. The results indicated that the growth and proliferation of RAW264.7 cells were promoted by RTW-1, and the number of cell divisions was enhanced, demonstrating a stimulatory effect.

##### Glycogen (PAS) Staining

**Figure 10:**
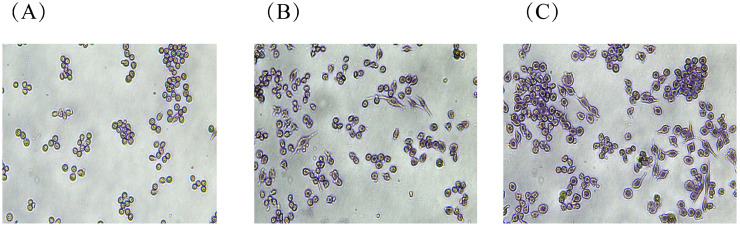
PAS staining of RAW264.7 with different concentrations of RTW-1; (**A**) blank control group; (**B**) 20 μg/mL RTW-1; (**C**) 80 μg/mL RTW-1.

Glycogen changes in RAW264.7 cells were observed using the Schiff method [65]. The results are shown in Figure 10A–C; untreated blank control cells exhibited a predominantly round morphology, were relatively fewer in number compared to groups B and C, and displayed relatively uniform size. This suggested that under unstimulated conditions, RAW264.7 cells maintain low glycogen content and relatively stable cell morphology. Following treatment with 20 μg/mL and 80 μg/mL RTW-1, as presented in Figure 10B,C, significant changes in cell morphology were observed compared to the blank control group. Specifically, nuclei were enlarged, and pseudopodia were extended by some cells, adopting irregular elongated spindle shapes. These alterations were more pronounced at the 80 μg/mL concentration. These results indicated that RTW-1 promoted RAW264.7 cell activation, thereby affecting glycogen metabolism and immune function.

### 3.14. Effects of RTW-1 on mRNA Expression Levels of Immune Factors in RAW264.7 Cells

To investigate the molecular mechanism underlying the immunomodulatory effects of *Rosa roxburghii* Tratt polysaccharide on macrophages, the mRNA expression of key immune factors in RAW264.7 cells was analyzed using qRT-PCR. One hallmark of macrophage function is cytokine production. Cytokines serve as critical regulators of physiological and pathological immune responses, with their expression directly reflecting the activation status of immune cells. Upon activation, increased levels of cytokines are secreted by macrophages, including TNF-α, IL-6, and IL-1β, all of which possess immunomodulatory activity [66]. Among these, tumor necrosis factor-α (TNF-α) is a key pro-inflammatory factor secreted by activated macrophages. It acts through autocrine mechanisms to enhance the production of various inflammatory mediators, thereby amplifying the immune response of monocytes and macrophages. Interleukin-1β (IL-1β) is a primary pro-inflammatory cytokine that promotes inflammatory responses and immune cell activation by activating signaling pathways such as NF-κB and p38 MAPK, while also inducing the expression of downstream factors like IL-6. IL-6 serves as a pivotal cytokine in inducing acquired immune responses. It promotes inflammatory reactions and Th17 cell differentiation while also exerting anti-inflammatory and tissue-repairing effects under certain conditions [67]. It plays a crucial role in facilitating T-cell and B-cell differentiation. IL-10, as an anti-inflammatory cytokine, suppresses excessive inflammatory responses and maintains immune homeostasis [68]. It is utilized to evaluate the immunoregulatory activity of macrophages.

**Figure 11:**
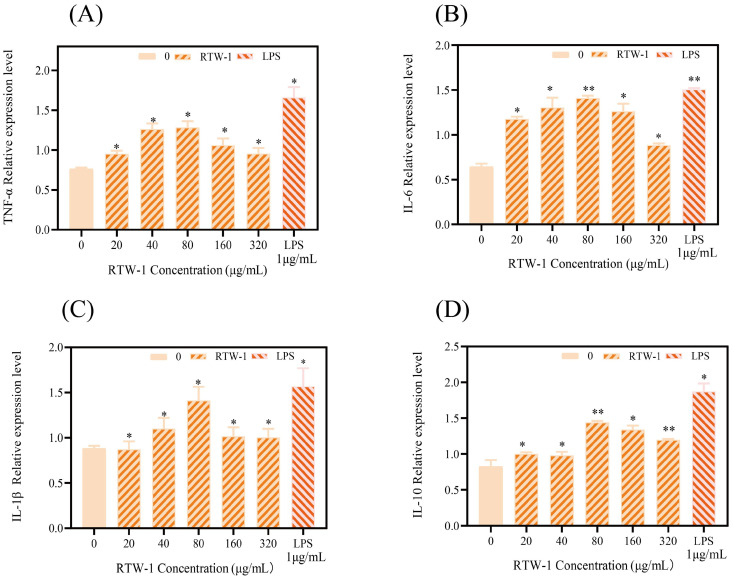
The effect of RTW-1 concentration on the relative expression level of TNF-α immune factor mRNA (**A**). The effect of RTW-1 concentration on the relative expression level of IL-6 immune factor mRNA (**B**). The effect of RTW-1 concentration on the relative expression level of IL-1β immune factor mRNA (**C**). The effect of RTW-1 concentration on the relative expression level of IL-10 immune factor mRNA (**D**) (* *p* < 0.05; ** *p* < 0.01; compared with the blank group, the difference was statistically significant).

As shown in Figure 11A–D, the relative expression of each cytokine increased in a concentration-dependent manner within the RTW-1 range of 10–80 μg/mL. At 80 μg/mL, expression levels peaked, reaching 85%, 141%, 90%, and 112% of the blank control levels, respectively, and approached those of the positive control. These results indicate that RTW-1 enhanced the expression of Th1-type cytokines (TNF-α, IL-1β, and IL-6) in RAW264.7 cells. The marked increase in IL-10 further suggested promotion of Th2-type response. Together, these effects enhanced the immunological activity of RAW264.7 cells.

### 3.15. Limitations of the Study

One limitation of this study is that endotoxin levels in the RTW-1 preparation were not determined. Although routine depyrogenation procedures were implemented, including the use of endotoxin-free water, heat treatment of glassware, and 0.22 µm filtration, the possibility of trace LPS contamination cannot be completely excluded. The observed immune responses showed a clear concentration-dependent pattern, which argues against LPS contamination as the primary driver. Future studies should include LAL testing or polymyxin B inhibition assays to confirm that the immunomodulatory activity is an intrinsic property of RTW-1.

## 4. Conclusions

This study isolated and purified an acidic polysaccharide RTW-1 with a molecular weight of 2.16 × 10^3^ kDa from *Rosa roxburghii* Tratt. Its FT-IR spectrum exhibited typical polysaccharide absorption peaks. The glycosidic linkage sequence of RTW-1 was determined through further analysis of monosaccharide composition and methylation. Furthermore, RTW-1 exhibited an esterification degree of 58.30%. Rheological analysis revealed shear-thinning behavior: as the concentration of RTW-1 increased, the apparent viscosity (η), thixotropic loop area, storage modulus (G′), and loss modulus (G″) all increased. This led to higher solution viscosity and suggested a potential to prolong intestinal retention time. Within the concentration range of 10–320 μg/mL, RTW-1 activated RAW264.7 cells, promoting macrophage proliferation and differentiation while enhancing phagocytic capacity. The optimal concentration for RTW-1 to enhance the immune activity of RAW264.7 cells was determined to be 80 μg/mL. Analysis of cellular immune factor mRNA expression levels indicated that RTW-1 promoted the production of TNF-α, IL-10, IL-1β, and IL-6, thereby further regulating immune cell growth. Further evidence demonstrated RTW-1’s potent immunomodulatory activity. These findings confirm that RTW-1, owing to its unique physicochemical properties and active expression, can be applied to repair Cy-induced intestinal mucosal damage and enhance intestinal immunity. Consequently, it holds promising potential as a prospective immunostimulant for further enhancing the body’s immune response.

## Figures and Tables

**Table 1:** Primer design table.

Gene	Superscript	Subscript
β-actin	CAGCAAGCAGGAGTACGATGA	GGGTGTAAAACGCAGCTCAGTA
TNF-α	ATGGCCTCCCTCTCATCAGT	TTTGCTACGACGTGGGCTAC
IL-6	TCCTACCCCAATTTCCAATGCT	TAACGCACTAGGTTTGCCGA
IL-10	TGCAGTGTGTATTGAGTCTGCT	CGGAGAGAGGTACAAACGAGG
IL-1β	TGCCACCTTTTGACAGTGATG	ATGTGCTGCTGCGAGATTTG

**Table 2:** Results of RTW-1 methylation analysis.

Retention Time	Methylation of Sugar Residues	Linkage Types	Major Mass Fragments	Relative Molar Ratio
6.195	6-O-Me1-Man*p*	→2,3,4)-Man*p*-(1→	43, 61, 87, 99, 115, 128, 145, 157, 171, 187, 217, 260	0.04
6.313	2,3-O-Me2-Ara*p*	→4)-Ara*p*-(1→	43, 59, 87, 102, 115, 118, 129, 142, 165, 173, 189	0.16
6.492	2,3,6-O-Me3-Gal*p*	→4)-GalA*p*-(1→	43, 59, 87, 99, 103, 118, 131, 142, 158, 173, 187, 200	0.74
8.86	2,3,4-O-Me3-Rha*p*	T-Rha*p*	43, 59, 72, 85, 102, 118, 131, 145, 162, 175	0.09
9.039	2,3,6-O-Me3-Glc*p*	→4)-Glc*p*-(1→	43, 59, 72, 87, 103, 111, 118, 129, 142, 159, 173, 203	1.00
9.095	1,2,3,4,5-O-Ac5-Xyl*p*	→2,3,4)-Xyl*p*-(1→	43, 71, 85, 99, 115, 128, 145, 159, 175, 187, 217, 243, 261	0.11

**Table 3:** Changes in reducing sugar content in in vitro simulated digestion.

Digestion Phase	Reaction Time (h)	Reducing Sugar (mg/mL)
Saliva	0	0.0395 ± 0.0100
	0.25	0.0413 ± 0.0100
	0.75	0.0409 ± 0.0100
	1	0.0421 ± 0.0100
	0	0.0447 ± 0.0100
Gastric Juice	2	0.0452 ± 0.0100
	4	0.0462 ± 0.0100
	6	0.0471 ± 0.0100
	0	0.0513 ± 0.0100
Intestinal Juice	2	0.0521 ± 0.0100
	4	0.0534 ± 0.0100
	6	0.0537 ± 0.0100

## Data Availability

The original contributions presented in this study are included in the article/Supplementary Material. Further inquiries can be directed to the corresponding author.

## Supplementary Materials

The following supporting information can be downloaded at: https://www.mdpi.com/article/10.3390/foods15101641/s1, Figure S1: Glucose Standard Curve; Figure S2: Galacturonic Acid Standard Curve; Table S1: Removal of genomic DNA reaction sample syste; Table S2: Reverse transcription reaction sample system; Table S3: Quantitative real-time polymerase chain reaction sample addition system; Table S4: Two-step PCR amplification conditions.
