# Supplementary material for: A Novel Hydrophilic Colloidal Polysaccharide from Rosa roxburghii Tratt: Structural Characterization, Rheological Behavior and Immunomodulatory Activity
Source: Foods. 2026 May 8;15(10):1641. doi: 10.3390/foods15101641 (PMC13205491; doi:10.3390/foods15101641)
Supplement: Supplementary file 1 [file foods-15-01641-s001.zip › foods-4252059-supplementary.pdf]

# A novel hydrophilic colloidal polysaccharide from *Rosa roxburghii Tratt*: Structural characterization, rheological behavior and immunomodulatory activity

Chenxi Cui[a,b,c,1], Miao Wang[a,b,c,1], Qiuli Zhang[a,b,c], Xinzhu Zhang[a,b,c], Qi zhu[a,b,c], Liuya Wang[a,b,c], Tengda Li[a,b,c], Zhenyuan Zhu[a,b,c,*]

[a]*State Key Laboratory of Food Nutrition and Safety, Tianjin University of Science and Technology, Tianjin 300457, P.R. China*

[b]*Key Laboratory of Food Nutrition and Safety, Ministry of Education, Tianjin University of Science and Technology, Tianjin 300457, P.R. China*

[c]*College of Food Science and Engineering, Tianjin University of Science and Technology, Tianjin 300457, P.R. China*

[1]*Chenxi Cui and Miao Wang contributed to the work equally and should be regarded as co-first authors.*

** Corresponding author : Tel.: +86 2260912390, Fax: +86 2260912390.*

*E-mail address: zhyuanzhu@tust.edu.cn. (Z.-Y. Zhu).*

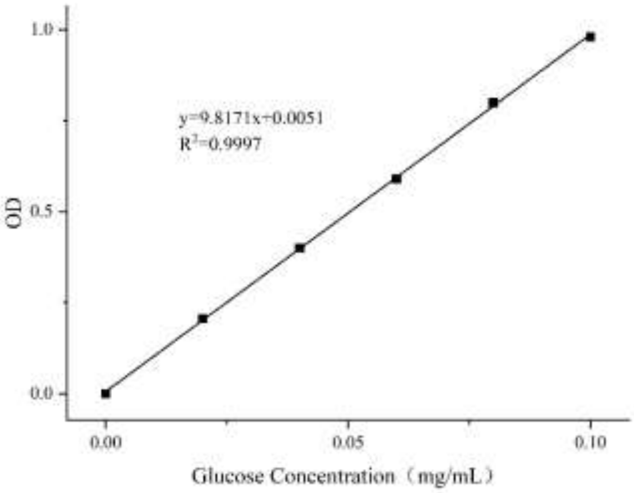


13

Figure S1. Glucose Standard Curve

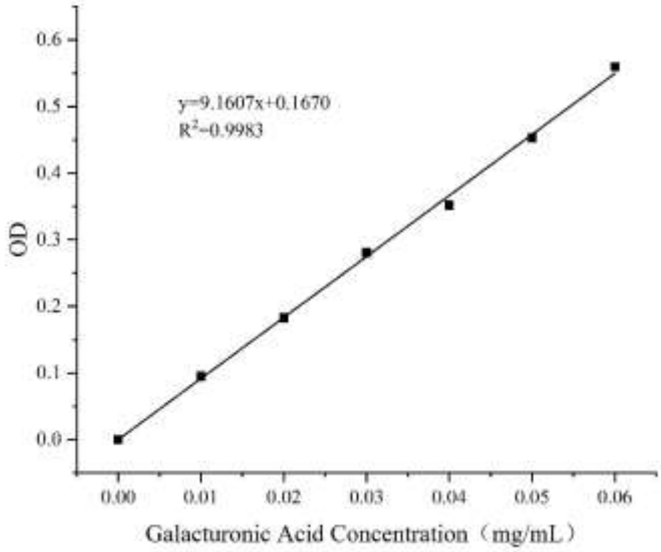


Figure S2. Galacturonic Acid Standard Curve

Table S1 Removal of genomic DNA reaction sample system

| **Reagent** | **Addition amount** |
|---|---|
| 5×gDNA Eraser Buffer | 2.0 μL |
| gDNA Eraser | 1.0 μL |
| Total RNA | Variable（1 ng-1 μg/rxn） |
| RNase Free $dH_2O$ | Add to 10 μL |

Table S2 Reverse transcription reaction sample system

| Reagent | Addition amount |
|---|---|
| Table S1 Reaction mixture | 10.0 μL |
| PrimeScript RT Enzyme Mix I | 1.0 μL |
| RT Primer Mix | 1.0 μL |
| 5×PrimeScript Buffer 2（for Real Time） | 4.0 μL |
| RNase Free $dH_2O$ | Add to 20 μL |

Table S3 Quantitative real-time polymerase chain reaction sample addition system

| Reagent | Addition amount |
|---|---|
| TB Green *Premix Ex Taq* II（Tli RNaseH Plus）（2X） | 10 μL |
| PCR Forward Primer（10 μM） | 0.4 μL |
| PCR Reverse Primer（10 μM） | 0.4 μL |
| Template DNA（<100 ng） | 2 μL |
| RNase Free $dH_2O$ | Add to 20 μL |

Table S4 Two-step PCR amplification conditions

| Step | Temperature | Duration | Cycles |
|---|---|---|---|
| pre-denaturation | 95°C | 30 sec | |
| PCR reaction | 95°C | 5 sec | 40 |
| Melt Curve | 60°C | 1 min | |

According to the manufacturer's instructions, after confirming the amplification curves and melting curves, the calculation was performed using the $2^{-\Delta\Delta CT}$ method.
